# HIV-1 drug resistance and genetic clustering among ART-treated individuals with virologic failure in Aksu, China

**DOI:** 10.3389/fmicb.2025.1622515

**Published:** 2026-01-16

**Authors:** Zhenzhen Dai, Hu Li, Mingyu Xu, JiangTao Feng, Liwen Sun, Di Lu, Yuxue Bi

**Affiliations:** 1 School of Medicine, Tarim University, Alar, China; 2 Aksu Prefecture Center for Disease Control and Prevention, Aksu, China; 3 College of Life Sciences and Technology, Tarim University, Alar, China; 4 School of Public Health, Xi’an Jiaotong University Health Science Center, Xi’an Jiaotong University Health Science Center, Xi’an, China

**Keywords:** antiretroviral drug resistance, antiretroviral therapy, cluster analysis, genetic network, HIV-1

## Abstract

**Background:**

Aksu Prefecture is among the regions most heavily affected by HIV-1 in China, yet data on acquired drug resistance (ADR) among antiretroviral therapy (ART)–treated individuals with virologic failure remain limited. This study aimed to characterize the prevalence, mutation patterns, and genetic clustering of drug resistance mutations (DRMs) in Aksu.

**Methods:**

We conducted a retrospective study among ART-treated individuals with virologic failure in Aksu Prefecture from 2022 to 2023. HIV-1 pol sequences were obtained from 675 individuals to identify DRMs. Genetic networks were constructed to assess clustering among individuals harboring DRMs (*n* = 407). Univariable and multivariable logistic regression analyses were used to identify factors associated with DRMs clustering.

**Results:**

The prevalence of ADR was 56.9% (384/675). CRF07_BC was the predominant subtype (97.6%). The most common DRMs were K103N/S (60.7%), M184V/I (27.3%), G190A/E/S (11.3%), and E138A/K/Q/G (10.8%), conferring high-level resistance mainly to lamivudine (3TC), efavirenz (EFV), and nevirapine (NVP). K65R was more frequent among individuals receiving TDF + 3TC + EFV/NVP, whereas Q58E was more common among those receiving LPV/r + 3TC + TDF/AZT (both *p* < 0.05). Genetic network analysis showed that 34.2% (139/407) of individuals with DRMs formed clusters. Higher viral load was associated with clustering, whereas LPV/r-based regimens were associated with a lower likelihood of clustering.

**Conclusion:**

HIV-1 ADR remains highly prevalent among ART-treated individuals with virologic failure in Aksu. Extensive resistance to NNRTIs was observed, whereas susceptibility to LPV/r was largely preserved. The clustering of DRMs underscores the importance of molecular surveillance for guiding targeted interventions. These findings support accelerating access to effective second-line regimens, strengthening pretreatment resistance surveillance, and prioritizing adherence support among central individuals with high viral loads.

## 1 Introduction

The global 95–95-95 strategy aims to eliminate HIV/AIDS as a public health threat by 2030 (UNAIDS, 2015). Antiretroviral therapy (ART) is central to this goal and has markedly reduced both HIV-related mortality and transmission (Gandhi et al., 2023). In 2016, China implemented a “Treat All” policy, which provides free ART to all people living with HIV (PLWH) regardless of CD4^+^ T-cell count. This policy has substantially improved national ART coverage, placing China among countries with high levels of ART coverage globally (Shen et al., 2016; Wu et al., 2019; Cao et al., 2020). However, the rapid scale-up of ART has also been accompanied by an increasing burden of antiretroviral drug resistance (ADR), which threatens the long-term effectiveness of first-line regimens (Cao et al., 2020; Haris and Rizwan, 2024). National surveillance data show that ADR prevalence increased markedly from 26.16% in 2003 to 56.16% in 2022 (Wang et al., 2024), highlighting the urgent need for strengthened resistance monitoring and adaptive treatment strategies.

Within this national context, the Xinjiang Uygur Autonomous Region remains one of the areas most heavily affected by HIV in China. Although several studies have reported ADR in Xinjiang, many are based on limited or outdated data. A survey conducted in 2018 reported an overall ADR prevalence of 50.44% in the region, with a markedly higher rate of 64.14% in Aksu Prefecture. Of particular concern was the protease inhibitor resistance rate of 5.58%, which exceeds levels typically observed in other parts of China and suggests a distinct local resistance profile (Wang et al., 2018). These findings underscore the need for updated and comprehensive studies to support targeted intervention strategies in this high-burden setting.

Since the first HIV case was reported in Aksu in 1996, the epidemic has expanded rapidly, with more than 10,000 cumulative reported cases by 2018. The epidemic was initially driven by injecting drug use but has shifted predominantly to heterosexual transmission since 2010 (Dai et al., 2020). As a prefecture in southern Xinjiang bordering Kyrgyzstan, Tajikistan, and Afghanistan, Aksu serves as an important hub linking China with Central Asia. Economic development has increased population mobility, including among people living with HIV (PLWH). Coupled with suboptimal treatment adherence, this mobility may increase the risk of HIV-1 variant dissemination, virological failure, and challenges to epidemic control (Shu et al., 2018). Despite these concerns, no large-scale study has specifically evaluated HIV drug resistance in Aksu, which limits the development of evidence-based and locally tailored intervention strategies.

Genetic network analysis has emerged as a powerful tool for identifying clusters of drug resistance mutations (DRMs), including among individuals with virological failure (Zhou et al., 2021; Xu et al., 2021; Kang et al., 2021). When integrated with resistance profiling and phylogenetic analysis, this approach enables the identification of clusters harboring DRMs, thereby supporting more focused and targeted public health responses (Chen et al., 2022; Yuan et al., 2020). To address existing knowledge gaps, this study aimed to determine the prevalence and mutation patterns of ADR among ART-treated individuals with virological failure in Aksu Prefecture during 2022–2023. Using genetic network analysis, we further sought to identify DRM clusters and examine associated factors to support treatment optimization and tailored public health strategies in this vulnerable region.

## 2 Materials and methods

### 2.1 Study participants

Between January 2022 and December 2023, a total of 1,048 ART-treated individuals with virologic failure were identified in Aksu Prefecture based on routine clinical monitoring. Virologic failure was defined as a confirmed HIV-1 diagnosis, at least 6 months of ART, and an HIV RNA level >1,000 copies/mL. Among these individuals, HIV-1 pol sequences were successfully obtained from 704 samples (67.2%), while sequencing failure in the remaining 344 samples was mainly due to low viral RNA levels, RNA degradation, or PCR amplification failure. If an individual experienced more than one episode of virologic failure during the study period, only the earliest eligible sample was included. All subsequent samples from the same individual were excluded to avoid duplicate representation. After removing duplicate samples, 675 ART-treated individuals with valid pol sequences constituted the final analytic population.

### 2.2 Data collection

Sociodemographic and clinical information was collected for all participants. Sociodemographic variables included sex, ethnicity, age, marital status, educational attainment, and mode of HIV transmission. Clinical data included duration of ART, current ART regimen, and drug resistance results, which were extracted from participants’ medical records.

### 2.3 Laboratory methods

Five milliliters of EDTA-anticoagulated whole blood was collected from each participant. One tube of whole blood was processed within 6 h for CD4^+^ T-lymphocyte testing at the local Centers for Disease Control and Prevention (CDCs), with analysis completed within 24 h. The remaining whole-blood samples were centrifuged at 3,000 rpm for 15 min, after which plasma was separated and stored at −80 °C. All plasma samples were subsequently transported under frozen conditions to the Aksu Regional Center for Disease Control and Prevention for HIV drug resistance testing.

### 2.4 HIV nucleic acid extraction, amplification, and sequencing

Viral RNA was extracted from plasma samples using the QIAamp Viral RNA Mini Kit (Qiagen, Germany). A nested PCR approach was used to amplify a 1.3-kb fragment of the HIV-1 pol region (corresponding to positions 2,166–3,462 of the HXB2 reference strain), according to a previously described protocol (Wang et al., 2018). PCR products were visualized by electrophoresis on a 1% agarose gel, purified, and sequenced by Beijing Dehongchangyuan Biotechnology Co., Ltd. Sequence fragments were assembled using Sequencher version 5.4.6.

### 2.5 HIV subtype analysis and drug resistance analysis

All sequences were aligned with HIV-1 reference sequences retrieved from the Los Alamos National Laboratory HIV Sequence Database using MEGA version 11.0.13 for multiple sequence alignment and preliminary phylogenetic analysis. HIV-1 subtypes were determined using both the HIV BLAST tool and the China HIV Gene Sequence Data Management and Analysis System^1^ (Feng et al., 2023). Drug resistance mutations were identified using the Stanford HIV Drug Resistance Database (HIVdb) algorithm (version 9.8, accessed in March 2025). All sequences were screened for APOBEC-mediated hypermutation using the Hypermut 2.0 tool and were further evaluated for sequence quality. No sequences were excluded based on these quality control procedures.

Resistance levels were assessed for a total of 20 antiretroviral drugs, including seven nucleoside reverse transcriptase inhibitors (NRTIs), five non-nucleoside reverse transcriptase inhibitors (NNRTIs), and eight protease inhibitors (PIs). The evaluated drugs comprised doravirine (DOR), efavirenz (EFV), nevirapine (NVP), etravirine (ETR), rilpivirine (RPV), fosamprenavir (FPV), abacavir (ABC), zidovudine (AZT), stavudine (D4T), didanosine (DDI), emtricitabine (FTC), lamivudine (3TC), tenofovir (TDF), atazanavir/ritonavir (ATV/r), darunavir/ritonavir (DRV/r), fosamprenavir/ritonavir (FPV/r), indinavir/ritonavir (IDV/r), nelfinavir (NFV), lopinavir/ritonavir (LPV/r), saquinavir/ritonavir (SQV/r), and tipranavir/ritonavir (TPV/r). Drug resistance was categorized into five levels based on the Stanford HIVdb scoring system: susceptible (0–9), potential low-level resistance (10–14), low-level resistance (15–29), intermediate-level resistance (30–59), and high-level resistance (≥60). Acquired drug resistance (ADR) was defined as the presence of at least one antiretroviral drug with a resistance score≥15, excluding cases classified solely as potential low-level resistance. ART-treated individuals with DRMs were defined as those harboring any mutation with a Stanford HIVdb resistance score≥10.

### 2.6 Phylogenetic analysis and genetic networks

An approximate maximum-likelihood (ML) phylogenetic tree was reconstructed using FastTree version 3.0, with reference sequences representing major HIV-1 subtypes included as outgroups. The generalized time-reversible model with gamma-distributed rate variation and a proportion of invariant sites (GTR + G + I) was applied for nucleotide substitution. Node support was evaluated using the Shimodaira-Hasegawa (SH) test implemented in the software. The resulting phylogenetic tree was further analyzed using Cluster Picker version 1.2.5 to identify genetic clusters, defined as clades with bootstrap support ≥95% and pairwise genetic distance ≤3%. All 675 sequences were included in the phylogenetic reconstruction. The final tree was visualized using iTOL version 6.

Pairwise genetic distances between sequences were calculated using HyPhy version 2.2.4 under the TN93 model, and the minimum genetic distance approach was applied to infer pairwise relationships. The TN93 genetic distance threshold for network construction was selected within the commonly accepted range for recent HIV transmission (0.5–1.5%), balancing sensitivity to ongoing transmission with control of spurious genetic linkages. The final threshold of 1.41% was applied for subsequent network analysis of DRMs clustering. Genetic networks were visualized using R version 4.4.2.

Network centrality metrics were defined in accordance with established methodologies (de Arruda et al., 2014; Little et al., 2014). Degree centrality was defined as the number of direct connections incident on a node, with nodes having a degree ≥3 classified as highly connected. Betweenness centrality quantified the extent to which a node lay on the shortest paths between other nodes, reflecting its role as a network bridge. Closeness centrality was calculated as the reciprocal of the sum of the shortest path distances from a node to all other reachable nodes, indicating the efficiency of information flow within the network. For both betweenness and closeness centrality, values >0.75 (normalized to a 0–1 scale) were considered high. Nodes exhibiting high values across all three centrality measures were designated as “central nodes” within the network (Zeng et al., 2021).

### 2.7 Statistical analysis

All statistical analyses were performed using R software. Categorical variables across different antiretroviral therapy regimens were compared using the chi-squared test to assess differences in HIV drug resistance mutation profiles. Factors associated with DRMs clustering were evaluated using univariable and multivariable logistic regression analyses. Variables with *p* < 0.10 in the univariable analyses, together with those of established clinical relevance, were entered into the initial multivariable model. A stepwise selection procedure was applied to derive the final multivariable model. Potential multicollinearity among covariates was assessed using variance inflation factors (VIFs), with all VIF values <5 indicating the absence of significant multicollinearity. Model fit was evaluated using the Hosmer–Lemeshow goodness-of-fit test. All statistical tests were two-tailed, and a *p* value < 0.05 was considered statistically significant.

## 3 Results

### 3.1 Characteristics of the study population

**Figure 1:**
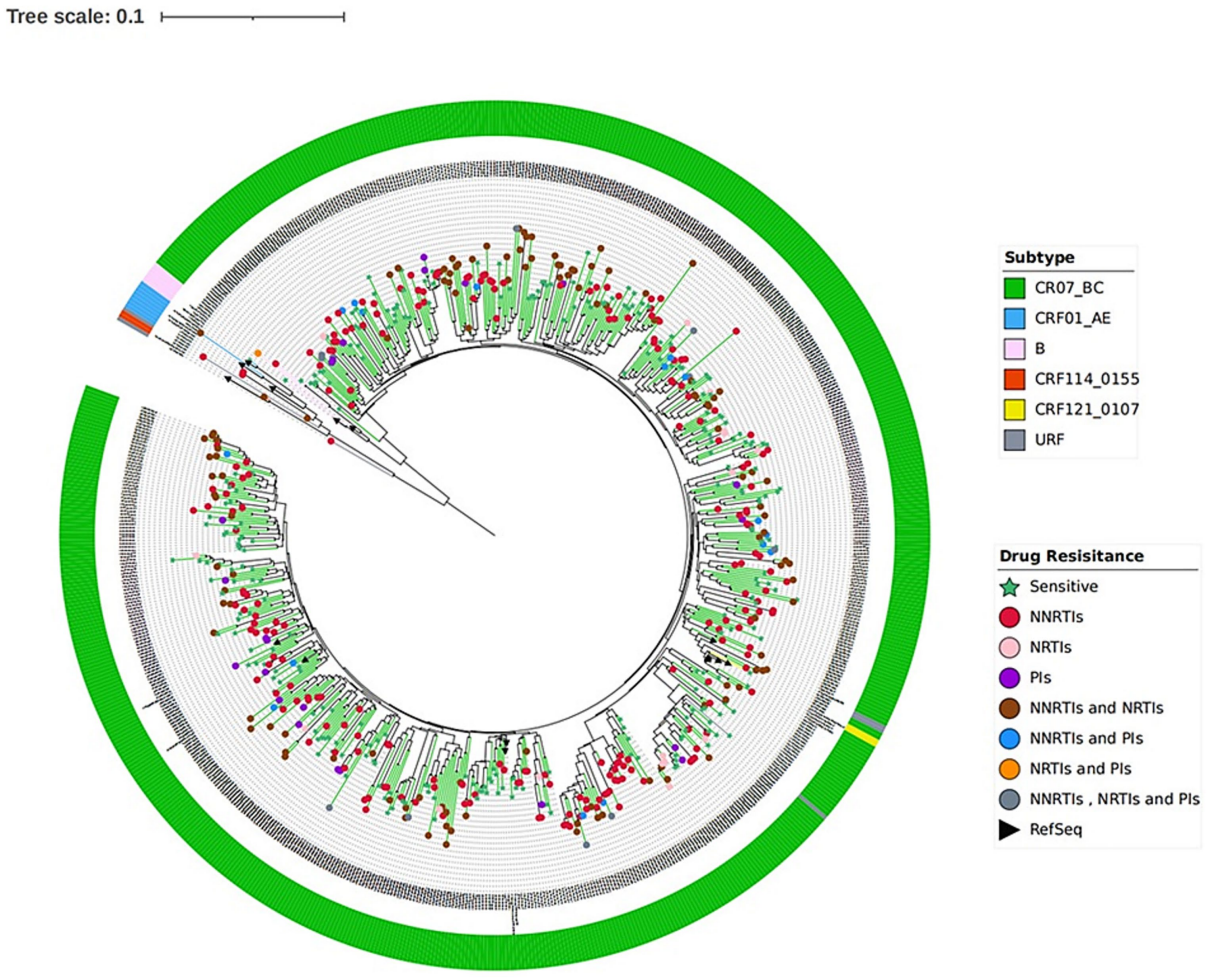
ML phylogenetic tree with 675 nucleotide sequences from ART-treated individuals with virological failure.

A total of 675 ART-treated individuals with virological failure were included in this study, of whom 398 (58.96%) were male and 277 (41.04%) were female. Most participants were aged 15–49 years, accounting for 77.19% (521/675) of the study population. The predominant route of HIV transmission was heterosexual contact, which accounted for 92.00% (621/675) of cases. Overall, 50.52% (341/675) of participants were married, and 94.37% (637/675) were of Uyghur ethnicity. Approximately 75.56% (510/675) of patients were receiving first-line ART, mainly TDF/AZT + 3TC + EFV/NVP–based regimens. More than half of the participants (55.11%, 372/675) had a viral load >10,000 copies/mL at the time of sampling (Supplementary Table S1). The median duration of ART was 58 months (interquartile range [IQR]: 38–83 months). CRF07_BC was the predominant HIV-1 genotype, accounting for 97.63% (659/675) of cases, whereas CRF01_AE was rare (0.74%, 5/675) (Supplementary Table S1 and Figure 1).

### 3.2 Analysis of DRMs

**Figure 2:**
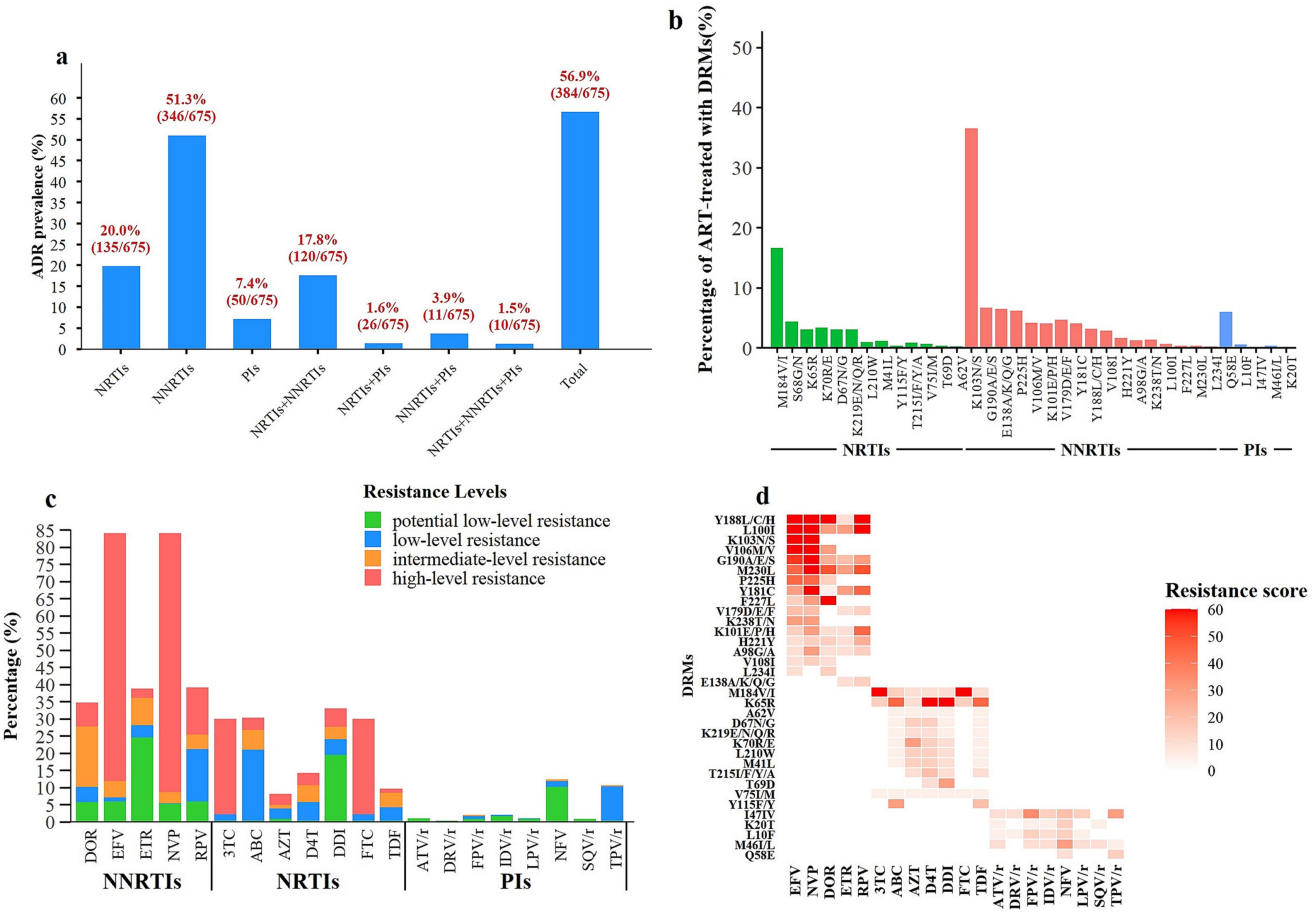
Prevalence and patterns of HIV-1 drug resistance among ART-treated individuals. **(a)** Prevalence of HIV-1 drug resistance by antiretroviral drug class. **(b)** Distribution of HIV-1 drug resistance mutations (DRMs). **(c)** Resistance levels of DRMs to different antiretroviral drugs. **(d)** Association between major antiretroviral drugs and key resistance mutation sites; darker colors indicate stronger effects of mutation sites on drug resistance. EFV, efavirenz; NVP, nevirapine; DOR, doravirine; ETR, etravirine; RPV, rilpivirine; 3TC, lamivudine; ABC, abacavir; AZT, zidovudine; d4T, stavudine; ddI, didanosine; FTC, emtricitabine; TDF, tenofovir disoproxil fumarate; ATV/r, atazanavir/ritonavir; DRV/r, darunavir/ritonavir; FPV/r, fosamprenavir/ritonavir; IDV/r, indinavir/ritonavir; LPV/r, lopinavir/ritonavir; NFV, nelfinavir; SQV/r, saquinavir/ritonavir; TPV/r, tipranavir/ritonavir.

Among the 675 ART-treated individuals with virological failure, 407 (60.2%) were found to harbor drug resistance mutations (DRMs). The overall prevalence of ADR was 56.9% (384/675) during 2022–2023 (Figure 2a). Resistance to NRTIs, NNRTIs, and PIs was observed in 20.0% (135/675), 51.3% (346/675), and 7.4% (50/675) of participants, respectively. Notably, dual-class resistance to both NRTIs and NNRTIs was observed in 17.8% (120/675) of participants (Figure 2a). A total of seven NRTI-associated, five NNRTI-associated, and eight PI-associated DRMs were identified. The most common NRTI-associated mutation was M184V/I (27.3%), followed by S68G/N (7.4%) and K70R/E (5.7%). The most prevalent NNRTI-associated mutation was K103N/S (60.7%), followed by G190A/E/S (11.3%), E138A/K/Q/G (10.8%), and P225H (10.3%). All PI-associated mutations were observed at low frequencies, except for Q58E (10.07%) (Table 1 and Figure 2b).

**Table 1:** Distribution of HIV-1 drug resistance mutations by antiretroviral regimen, *n* (%).*

Mutation	DRMs	TDF + 3TC + EFV/NVP (*n* = 226)	AZT + 3TC + EFV/NVP (*n* = 85)	LPV/r + 3TC + TDF/AZT (n = 93)	Other (*n* = 3)	total (*n* = 407)	*χ^2^*	*P*
Resistance to NRTIs	M184V/I	60(26.55)	28(32.94)	24(25.81)	1(33.3)	113(27.76)	0.752	0.687
	S68G/N	21(9.29)	7(8.24)	2(2.15)	0(0.00)	30(7.37)	4.386	0.112
	K70R/E	13(5.75)	2(2.35)	7(7.53)	1(33.3)	23(5.65)	4.689	0.096
	K65R	18(7.96)	2(2.35)	1(1.08)	0(0.00)	21(5.16)	6.494	**0.039**
	D67N/G	11(4.87)	2(2.35)	7(7.53)	1(33.3)	21(5.16)	4.891	0.087
	K219E/N/Q/R	9(3.98)	4(4.71)	8(8.60)	0(0.00)	21(5.16)	2.058	0.357
	L210W	4(1.77)	2(2.35)	1(1.08)	0(0.00)	7(1.72)	0.497	0.780
	M41L	4(1.77)	1(1.18)	3(3.23)	0(0.00)	8(1.97)	0.879	0.644
	Y115F/Y	3(1.33)	0(0.00)	0(0.00)	0(0.00)	3(0.74)	1.047	0.592
	T215I/F/Y/A	2(0.88)	1(1.18)	3(3.23)	0(0.00)	6(1.47)	2.363	0.307
	V75I/M	2(0.88)	0(0.00)	3(3.23)	0(0.00)	5(1.23)	3.503	0.173
	T69D	1(0.44)	0(0.00)	2(2.15)	0(0.00)	3(0.74)	2.694	0.260
	A62V	0(0.00)	1(1.18)	1(1.08)	0(0.00)	2(0.49)	3.157	0.206
Resistance to NNRTIs	K103N/S	130(57.52)	56(65.88)	59(63.44)	2(66.7)	247(60.69)	1.377	0.502
	G190A/E/S	28(12.39)	8(9.41)	10(10.75)	0(0.00)	46(11.3)	0.384	0.825
	E138A/K/Q/G	27(11.95)	4(4.71)	13(13.98)	0(0.00)	44(10.81)	3.769	0.152
	P225H	24(10.62)	9(10.59)	9(9.68)	0(0.00)	42(10.32)	0.000	1.000
	V106M/V	22(9.73)	4(4.71)	3(3.23)	0(0.00)	29(7.13)	4.585	0.101
	K101E/P/H	20(8.85)	2(2.35)	6(6.45)	0(0.00)	28(6.88)	3.105	0.212
	V179D/E/F	16(7.08)	11(12.94)	5(5.38)	0(0.00)	32(7.86)	2.650	0.266
	Y181C	15(6.64)	3(3.53)	9(9.68)	1(33.3)	28(6.88)	4.476	0.107
	Y188L/C/H	13(5.75)	5(5.88)	4(4.30)	0(0.00)	22(5.41)	0.144	0.931
	V108I	9(3.98)	5(5.88)	6(6.45)	0(0.00)	20(4.91)	0.886	0.642
	H221Y	7(3.10)	3(3.53)	2(2.15)	0(0.00)	12(2.95)	0.151	0.927
	A98G/A	6(2.65)	1(1.18)	2(2.15)	0(0.00)	9(2.21)	0.203	0.904
	K238T/N	6(2.65)	3(3.53)	1(1.08)	0(0.00)	10(2.46)	1.027	0.598
	L100I	5(2.21)	0(0.00)	0(0.00)	0(0.00)	5(1.23)	2.640	0.267
	F227L	3(1.33)	0(0.00)	0(0.00)	0(0.00)	3(0.74)	1.052	0.591
	M230L	3(1.33)	0(0.00)	0(0.00)	0(0.00)	3(0.74)	1.061	0.588
	L234I	2(0.88)	0(0.00)	0(0.00)	0(0.00)	2(0.49)	0.000	1.000
Resistance to PIs	Q58E	14(6.19)	11(12.94)	16(17.2)	0(0.00)	41(10.07)	8.103	**0.017**
	L10F	2(0.88)	0(0.00)	2(2.15)	0(0.00)	4(0.98)	1.321	0.517
	I47IV	1(0.44)	0(0.00)	0(0.00)	0(0.00)	1(0.25)	0.000	1.000
	M46I/L	1(0.44)	1(1.18)	1(1.08)	0(0.00)	3(0.74)	1.746	0.418
	K20T	0(0.00)	0(0.00)	1(1.08)	0(0.00)	1(0.25)	1.579	0.454

*Drug resistance mutations (DRMs) were defined as mutations with a Stanford HIVdb resistance score≥10, including potential low-level resistance. Bold type indicates statistical significance.

### 3.3 The drug susceptibility of sequences with DRMs

Drug susceptibility was evaluated for 20 antiretroviral agents among sequences harboring any drug resistance mutations (DRMs), including NRTIs, NNRTIs, and PIs. High-level resistance to EFV and NVP was observed in 72.5 and 75.7% of sequences, respectively. Within the NRTI class, high-level resistance to FTC and 3TC was more prevalent than that to other NRTIs (Figure 2c). The primary NRTI resistance mutation M184V/I conferred high-level resistance to 3TC and FTC, whereas K65R was associated with intermediate-level resistance to abacavir (ABC) and tenofovir disoproxil fumarate (TDF). The most frequent NNRTI-associated resistance mutations included K103N/S, V106M/V, and G190A/E/S, which conferred high-level resistance to both NVP and EFV (Figure 2d).

### 3.4 Distribution of DRMs by ART regimen

DRMs were detected across all ART regimens examined. Among the 12 major NRTI-associated mutations, 11 were identified in both the TDF + 3TC + EFV/NVP and LPV/r + 3TC + TDF/AZT regimens, and 10 were observed in the AZT + 3TC + EFV/NVP regimen, whereas only three were detected in other regimens. The K65R mutation, which is associated with reduced susceptibility to TDF, was significantly more frequent among individuals receiving the TDF + 3TC + EFV/NVP regimen (*p* < 0.05). The K103N/S mutation showed the highest overall prevalence across all regimens. PI-associated DRMs were generally uncommon; however, Q58E was more frequently observed in the LPV/r + 3TC + TDF/AZT regimen, despite not typically conferring high-level resistance to LPV/r.

### 3.5 Genetic networks analysis of ART-treated individuals with DRMs

**Figure 3:**
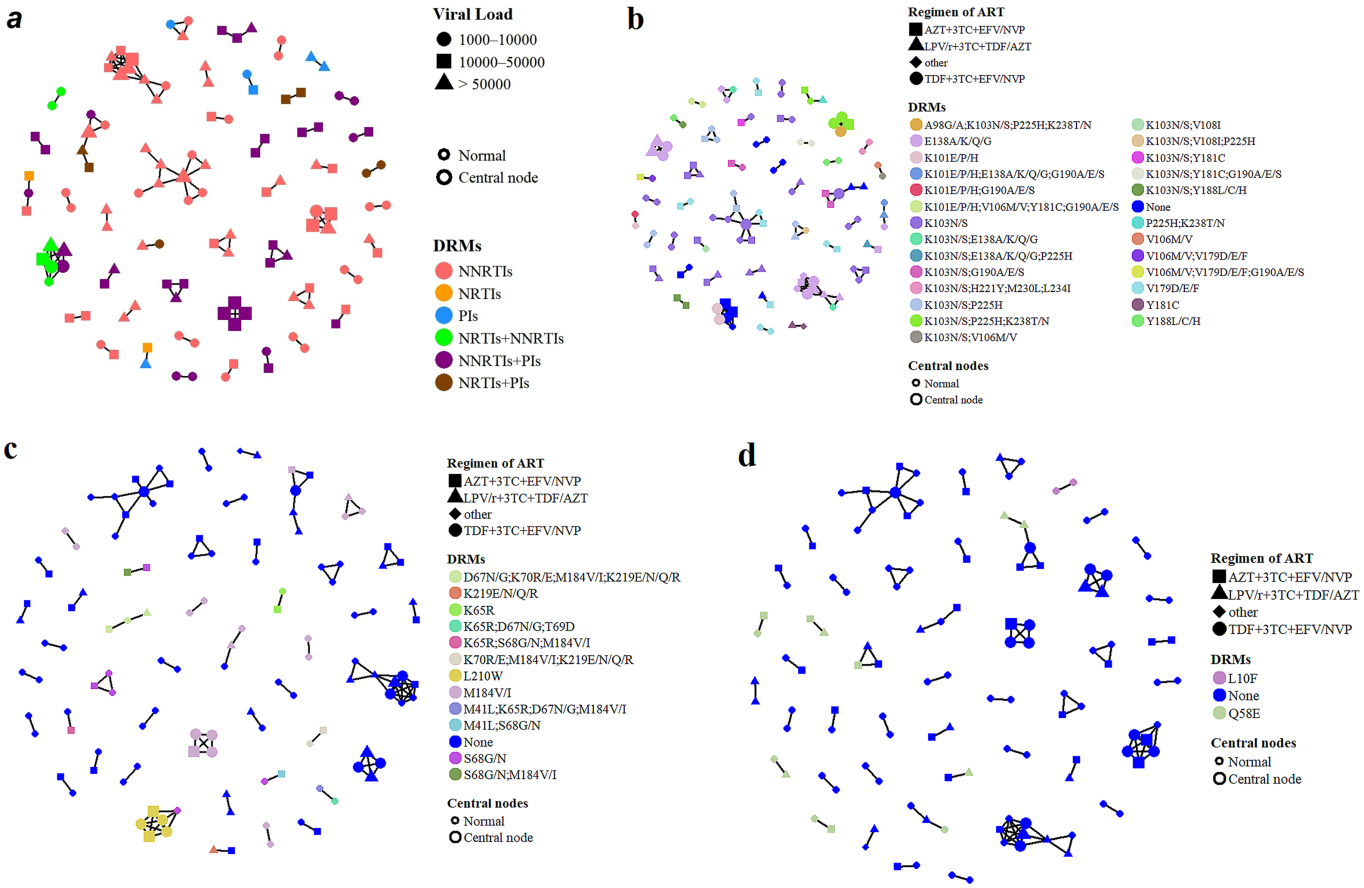
Genetic network of HIV-1 DRMs and centrality analysis among ART-treated individuals. **(a)** Overall molecular network of HIV-1 DRMs showing node centrality and viral load levels. Node shapes represent different viral load categories, and outlined nodes indicate central nodes. Colors indicate different classes of DRMs. **(b)** Distribution of NNRTI-associated DRMs across ART regimens within the HIV-1 genetic network. **(c)** Distribution of NRTI-associated DRMs across ART regimens within the HIV-1 genetic network. **(d)** Distribution of PI-associated DRMs across ART regimens within the HIV-1 genetic network.

All 407 pol sequences harboring DRMs were included in the genetic network analysis using a TN93 genetic distance threshold of 1.41%. In total, 139 sequences (34.2%) formed 45 clusters, including 32 dyads and 13 clusters containing three or more sequences (Figure 3). After excluding sequences with only potential low-level resistance (score 10–14), 30 clusters containing clinically relevant DRMs (score ≥15) remained. Most clusters involved NNRTI-associated DRMs, with K103N/S, E138A/K/Q/G, V179D/E/F, and G190A/E/S being the most frequently observed. Among NRTI-associated DRMs, M184V/I and S68G/N predominated, whereas Q58E and L10F were the major PI-associated DRMs.

Centrality analysis was restricted to clusters containing at least three sequences. Within these clusters, 14.4% of nodes exhibited a degree centrality ≥3. A total of 18 central nodes were distributed across six genetic clusters, of which 10 harbored dual-class resistance mutations. Most central nodes (16/18) had viral loads >10,000 copies/mL (Figure 3a). Among NNRTI-associated DRMs, K103N/S was the most prevalent mutation (35 clusters), followed by E138A/K/Q/G and V179D/E/F (each in eight clusters), and G190A/E/S (seven clusters). Five central nodes harbored compound mutations, including K103N/S, P225H, and K238T/N (Figure 3b). M184V/I was the predominant NRTI-associated DRM (13 clusters), followed by S68G/N (seven clusters). Two major mutation clusters associated with central nodes involved L210W and M184V/I (Figure 3c). Among PI-associated DRMs, Q58E and L10F were detected within the network. L10F was observed only among individuals receiving first-line regimens, whereas Q58E was identified in patients failing both first- and second-line ART. This pattern suggests the possible presence of Q58E in clustered viruses prior to treatment initiation rather than acquisition solely under selective drug pressure (Figure 3d).

### 3.6 Risk factors for clustering of DRMs

**Figure 4:**
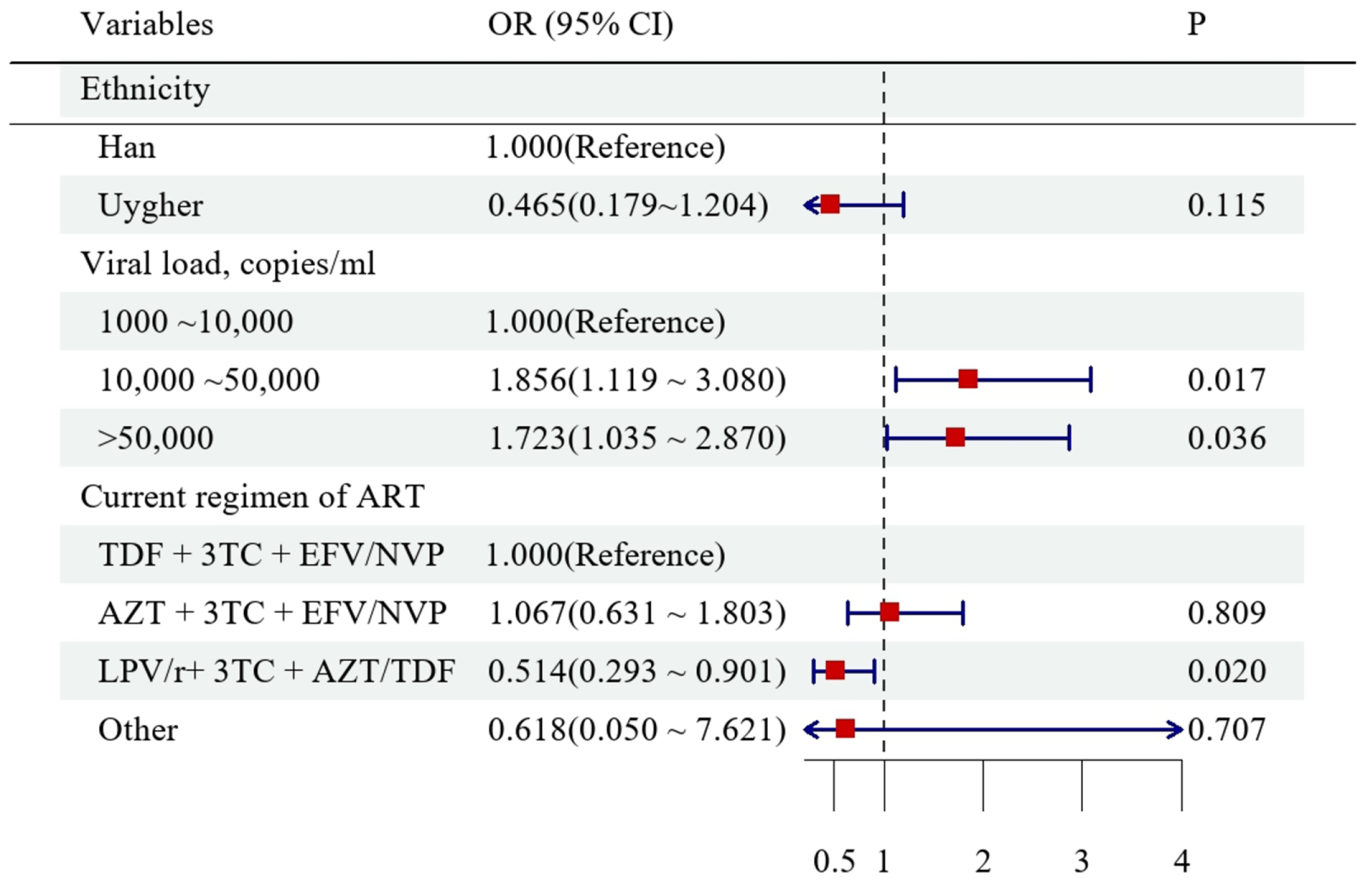
Multivariate analysis of factors associated with clustering of DRMs.

To identify factors associated with DRMs clustering, 14 independent variables were assessed using logistic regression analysis (Supplementary Table S2). Univariable analysis showed that ethnicity, viral load level, and current ART regimen were significantly associated with membership in a DRMs cluster (*p* < 0.05). In contrast, sex, age, marital status, educational level, transmission route, latest CD4^+^ T-cell count, duration of ART, presence of NRTI-, NNRTI-, or PI-associated DRMs, and viral subtype were not significantly associated with clustering (all *p* > 0.05; Supplementary Table S2). In the multivariable analysis, individuals with viral loads of 10,000–50,000 copies/mL (OR = 1.856, 95% CI:1.119–3.080) and>50,000 copies/mL (OR = 1.723, 95% CI: 1.035–2.870) had significantly higher odds of belonging to a DRMs cluster. In contrast, participants receiving the LPV/r + 3TC + AZT/TDF regimen were significantly less likely to belong to DRMs clusters (Figure 4).

## 4 Discussion

This study retrospectively analyzed acquired drug resistance (ADR) among people living with HIV who experienced virological failure in Aksu Prefecture from 2022 to 2023. The observed ADR prevalence (56.9%) exceeded the national pooled estimate (44.96%) as well as the rates reported in Shanghai (48%) and Hangzhou (51.33%), while being comparable to those in Liangshan (57.4%) and Jilin (58.6%) (Wang et al., 2024; Zhang et al., 2024a; Zhang et al., 2024b; Zhu et al., 2024; Pei et al., 2025; Qi et al., 2023). This pattern highlights pronounced within-country heterogeneity in the burden of ADR, likely driven by regional disparities in socioeconomic conditions and access to healthcare services.

At the global level, the high ADR prevalence observed in Aksu is consistent with patterns reported in resource-limited settings such as India, where prolonged reliance on NNRTI- and NRTI-based regimens remains common (Karade et al., 2018). A distinctive local feature of the Aksu epidemic is its extraordinary virological homogeneity, with the CRF07_BC subtype accounting for nearly all infections (97.63%). Different viral genetic backgrounds may exhibit varying propensities for specific DRM pathways (Sui et al., 2014; Yuan et al., 2015). In contrast to the greater subtype diversity observed in many high-ADR regions globally, such as parts of sub-Saharan Africa and South America, this uniform genetic background suggests a more constrained evolutionary landscape for resistance development and clustering in this setting (Hauser et al., 2022; Sno et al., 2021).

The ADR pattern observed in Aksu is consistent with the historical use of first-line antiretroviral regimens in China. NNRTI- (51.3%) and NRTI-associated resistance (20.0%) predominated, in line with national reports and reflecting the long-term reliance on efavirenz (EFV)- and nevirapine (NVP)-based regimens (Zhou et al., 2021; World Health Organization, 2023). The high frequencies of K103N/S and G190A/E/S among NNRTIs and M184V/I among NRTIs are typical consequences of this treatment background and suggest limited regimen updating over time, a pattern also reported in other middle-income settings. Although dolutegravir (DTG) has been nationally recommended as the preferred first-line regimen (National Center for AIDS/STD Control and Prevention, 2023), its implementation in Aksu remains extremely limited. In our study population, only 2 of 675 individuals (<1%) had received DTG, reflecting that DTG has not yet been incorporated into the routine free ART program because of local economic constraints. This gap between national policy and local implementation likely contributes to the continued reliance on NNRTI-based regimens and the persistence of a high ADR burden in this setting.

NNRTI-associated mutations K103N/S, V106M/V, and G190A/E/S confer high-level resistance to EFV and NVP, which remain core components of free first-line ART regimens in China. Although E138A/K/Q/G mutations confer only low-level resistance to rilpivirine (RPV) and doravirine (DOR), their increasing prevalence is concerning because of the potential impact on the antiviral activity of these alternative NNRTIs. The M184V/I mutation, selected by lamivudine (3TC) or emtricitabine (FTC), confers high-level resistance to 3TC and intermediate resistance to FTC, leading to substantial loss of antiviral efficacy. In Aksu Prefecture, where ART adherence has been reported to be low (46.26%) (Dai et al., 2021), the high frequency of M184V/I is consistent with the local treatment context. The K65R mutation, which reduces susceptibility to tenofovir (TDF), was more common among individuals receiving TDF + 3TC + EFV/NVP regimens, reflecting regimen-specific selective pressure (Cox et al., 2023); patients with TDF intolerance are accordingly switched to zidovudine (AZT)-based regimens in routine practice (Dinesha et al., 2016). Among PI-associated mutations, L10F was linked to low-level resistance to LPV/r, whereas Q58E did not appear to substantially impair LPV/r susceptibility.

Phylogenetic clusters containing three or more sequences represent groups of closely related viral lineages and are commonly used as proxies for putative transmission networks. Across major clusters, we identified several key DRMs, including K103N/S, P225H, K238T/N, E138A/K/Q/G, M184V/I, L210W, and Q58E, despite heterogeneous ART exposure histories. Mutations such as K103N/S and Q58E have been reported to retain relatively preserved viral replicative capacity, which may contribute to their persistence within clustered viral lineages under suboptimal drug pressure (Wertheim et al., 2017; Stekler et al., 2018). The detection of Q58E among individuals receiving first-line regimens further suggests the possible presence of baseline resistance within clustered populations, highlighting the importance of pretreatment drug resistance surveillance to guide regimen selection.

Centrality analysis identified key individuals (“central nodes”) that were associated with the aggregation of DRMs within clusters (Zhou et al., 2021; Zeng et al., 2021). Among the 139 sequenced cases, 20 (14.4%) occupied central positions. The enrichment of individuals with viral loads >10,000 copies/mL within clusters indicates that incomplete viral suppression is associated with both resistance accumulation and the expansion of cluster-related network structure. These findings reinforce that durable viral suppression achieved through effective ART is essential for limiting further growth of DRMs clusters. The presence of a limited number of highly central individuals further suggests that DRMs clustering within this network is structured around specific key nodes rather than being randomly distributed.

This network structure has important implications for clinical management and public health practice. Patients occupying central positions may be prioritized for intensified viral load monitoring, early resistance testing, and individualized adherence support. Improving viral suppression in this subgroup is expected to contribute to the containment of DRMs clustering at the network level. In addition, the lower clustering probability observed among patients receiving LPV/r-based regimens suggests that appropriate optimization of second-line ART is associated with a reduced likelihood of participation in DRMs clusters. Collectively, these findings support the integration of molecular network analysis into routine HIV care to guide targeted monitoring, rational regimen adjustment, and focused adherence interventions for the control of DRMs clustering.

This study has several limitations. First, the cross-sectional design, lack of diagnosis dates, and absence of detailed temporal epidemiological data limited longitudinal inference of resistance evolution and transmission dynamics. Second, DRM clustering was inferred from partial pol sequences using a genetic network based on a TN93 threshold, which identifies close viral relatedness but does not allow causal inference of transmission. Third, as the study was restricted to ART-treated individuals with virologic failure, pretreatment and transmitted drug resistance in untreated populations could not be assessed, and the findings mainly reflect acquired resistance. In addition, the incomplete sequencing rate and exclusion of individuals without successful sequencing may introduce selection bias. Despite these limitations, this study provides important region-specific insights into the burden and molecular clustering of acquired HIV-1 drug resistance in Aksu. Data availability statement.

## Data Availability

The HIV-1 pol sequence data generated in this study constitute human pathogen genetic data derived from human participants and are subject to access restrictions under local regulatory requirements and policies of the Aksu Prefecture Center for Disease Control and Prevention (CDC); therefore, public deposition in international sequence repositories (e.g., GenBank) is not permitted. De-identified sequence data may be made available to qualified researchers upon reasonable request and following formal review and approval by the Aksu Prefecture CDC and the relevant institutional ethics committee. Requests should include a clear scientific rationale and a description of the intended data use, and should be directed to Dr. H.L., Aksu Prefecture CDC (email: AKsuCDClihu@163.com).

## Footnotes

1
https://nmdc.cn/hiv/tool/sequence
