# Supplementary figures and images for: HIV-1 drug resistance and genetic clustering among ART-treated individuals with virologic failure in Aksu, China
Source: Front Microbiol. 2026 Jan 16;16:1622515. doi: 10.3389/fmicb.2025.1622515 (PMC12855523; doi:10.3389/fmicb.2025.1622515)

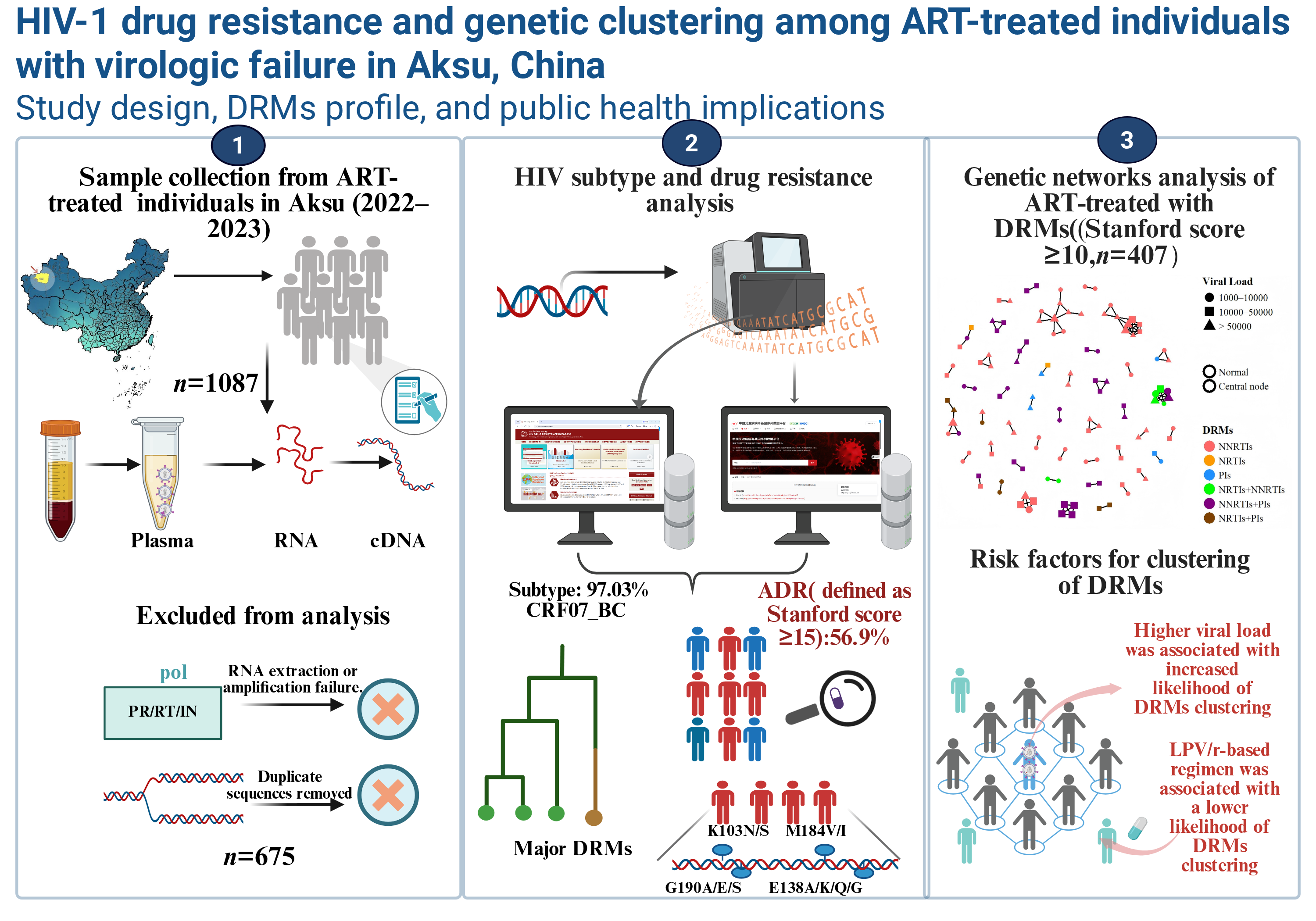

Supplement: Supplementary file 1 [file Image_1.PNG]
