# Supplementary material for: HIV-1 drug resistance and genetic clustering among ART-treated individuals with virologic failure in Aksu, China
Source: Front Microbiol. 2026 Jan 16;16:1622515. doi: 10.3389/fmicb.2025.1622515 (PMC12855523; doi:10.3389/fmicb.2025.1622515)
Supplement: Supplementary file 2 [file Table_1.DOC]

Table S1. Demographic characteristics of ART-treated individuals with virological failure in Aksu Prefecture from 2022 to 2023

| Variables | Number,*n* (%) | ART-treated individuals with DRMs , *n* (%)^*^ |
| --- | --- | --- |
| Total | 675(100) | 407(100) |
| Gender |  |  |
| Male | 398(58.96) | 239(58.72) |
| Female | 277(41.04) | 168(41.28) |
| Age |  |  |
| ≤15 | 18(2.67) | 16(3.93) |
| 15～49 | 521(77.19) | 318(78.13) |
| ≥50 | 136(20.15) | 73(17.94) |
| Ethnicity |  |  |
| Han | 38(5.63) | 19 (4.67) |
| Uygher | 637(94.37) | 388 (95.33) |
| Marital status |  |  |
| Single | 104(15.41) | 65 (15.97) |
| Married or living With spouse | 341(50.52) | 197 (48.40) |
| Divorced/widowed | 230(34.07) | 145 (35.63) |
| Education |  |  |
| Illiteracy | 27(4.00) | 17 (4.18) |
| Primary school | 220(32.59) | 138 (33.91) |
| Middle school | 333(49.33) | 196 (48.16) |
| High school or technical | 55(8.15) | 32 (7.86) |
| Junior college or above | 40(5.93) | 24 (5.90) |
| Transmission route |  |  |
| Heterosexual behaviors | 621(92.00) | 373 (91.65) |
| Man who have sex with man | 18(2.67) | 9 (2.21) |
| Drug injection | 15(2.22) | 8 (1.97) |
| Mother to child | 19(2.81) | 17 (4.18) |
| Unknown | 2(0.30) | 0 (0.00) |
| Year |  |  |
| 2022 | 297(44.00) | 137 (33.66) |
| 2023 | 378(56.00) | 270 (66.34) |
| Latest CD4^+^ cell count, cell/μl |  |  |
| ≤ 200 | 125(18.52) | 85 (20.88) |
| 201~500 | 369(54.67) | 184 (45.21) |
| >500 | 181(26.81) | 138 (33.91) |
| Time of ART, months |  |  |
| ≤ 12 | 44(6.52) | 22 (5.41) |
| 12~36 | 112(16.59) | 71 (17.44) |
| >36 | 519(76.89) | 314 (77.15) |
| Current regimen of ART |  |  |
| TDF + 3TC + EFV/NVP | 364(53.92) | 226 (55.53) |
| AZT + 3TC + EFV/NVP | 146(21.63) | 85 (20.88) |
| LPV/r+ 3TC + AZT/TDF | 159(23.56) | 93 (22.85) |
| Other | 6(0.89) | 3 (0.74) |
| Viral Load,copies/ml |  |  |
| 1000 ~10,000 | 303(44.89) | 182 (44.72) |
| 10,000 ~50,000 | 199(29.48) | 116 (28.50) |
| >50,000 | 173(25.63) | 109 (26.78) |
| Subtype |  |  |
| CRF07_BC | 659(97.63) | 396 (97.30) |
| CRF01_AE | 5(0.74) | 4 (0.98) |
| B | 4(0.59) | 2 (0.49) |
| other | 2(0.30) | 5 (1.23) |
| URF | 5(0.74) | 396 (97.30) |

^*^ART-treated with DRMs (including individuals with potential low-level resistance)

Table S2. Univariable analysis of factors associated with DRMs clustering

| Variables | Total(%) | In cluster(%) | Not in cluster(%) | *OR* | *P* |
| --- | --- | --- | --- | --- | --- |
| Gender |  |  |  |  |  |
| Male | 239 (58.72) | 82 (58.99) | 157 (58.58) | 1.000 (Reference) |  |
| female | 168 (41.28) | 57 (41.01) | 111 (41.42) | 0.983(0.648 -11.125) | 0.936 |
| Age, years |  |  |  |  |  |
| ≤15 | 14 (3.44) | 3 (2.16) | 11 (4.10) | 1.000 (Reference) |  |
| 15～49 | 320 (78.62) | 104 (74.82) | 216 (80.60) | 1.765 (0.482 -6.464) | 0.391 |
| ≥50 | 73 (17.94) | 32 (23.02) | 41 (15.30) | 2.862 (0.736 -11.125) | 0.129 |
| Marital status |  |  |  |  |  |
| Single | 65 (15.97) | 25 (17.99) | 40 (14.93) |  |  |
| Married or living With spouse | 197 (48.40) | 70 (50.36) | 127 (47.39) | 0.882(0.494~1.573) | 0.670 |
| Divorced/widowed | 145 (35.63) | 44 (31.65) | 101 (37.69) | 0.697(0.378~1.286) | 0.248 |
| Ethnicity |  |  |  |  |  |
| Han | 19 (4.67) | 10 (7.19) | 9 (3.36) | 1.000 (Reference) |  |
| Uygher | 388 (95.33) | 129 (92.81) | 259 (96.64) | 0.448 (0.178 -1.130) | 0.089* |
| Education level |  |  |  |  |  |
| Illiteracy | 17 (4.18) | 6 (4.32) | 11 (4.10) | 1.000 (Reference) |  |
| Primary school | 138 (33.91) | 39 (28.06) | 99 (36.94) | 0.722 (0.250 -2.088) | 0.548 |
| Middle school | 196 (48.16) | 68 (48.92) | 128 (47.76) | 0.974 (0.345 -2.748) | 0.960 |
| High school or technical | 32 (7.86) | 17 (12.23) | 15 (5.60) | 2.078 (0.618 -6.989) | 0.237 |
| Junior college or above | 24 (5.90) | 9 (6.47) | 15 (5.60) | 1.100 (0.302 -4.009) | 0.885 |
| Route of transmission |  |  |  |  |  |
| Heterosexual behaviors | 373 (91.65) | 126 (90.65) | 247 (92.16) | 1.000 (Reference) |  |
| Man who have sex with man | 9 (2.21) | 5 (3.60) | 4 (1.49) | 2.450 (0.647 -9.285) | 0.187 |
| Drug injection | 8 (1.97) | 4 (2.88) | 4 (1.49) | 1.960 (0.482 -7.969) | 0.347 |
| Mother to child | 17 (4.18) | 4 (2.88) | 13 (4.85) | 0.603 (0.193 -1.888) | 0.385 |
| Latest CD4^+^ cell count, cell/μl |  |  |  |  |  |
| ≤ 200 | 85 (20.88) | 52 (19.40) | 33 (23.74) | 1.000 (Reference) |  |
| 201~500 | 184 (45.21) | 120 (44.78） | 64 (46.04) | 0.840 (0.494 -1.430) | 0.521 |
| >500 | 138 (33.91) | 96 (35.82) | 42 (30.22) | 0.689 (0.391 -1.216) | 0.199 |
| Viral load, copies/ml |  |  |  |  |  |
| 1000 ~10,000 | 182 (44.72) | 50 (35.97) | 132 (49.25) | 1.000 (Reference) |  |
| 10,000 ~50,000 | 116 (28.50) | 46 (33.09) | 70 (26.12) | 1.735 (1.058 -2.844) | **0.029*** |
| >50,000 | 109 (26.78) | 43 (30.94) | 66 (24.63) | 1.720 (1.040 -2.846) | **0.035*** |
| Time of ART, months |  |  |  |  |  |
| ≤ 12 | 22 (5.41) | 10 (7.19) | 12 (4.48) | 1.000 (Reference) |  |
| 12~36 | 71 (17.44) | 26 (18.71) | 45 (16.79) | 0.693 (0.263 -1.826) | 0.458 |
| >36 | 314 (77.15) | 103 (74.10) | 211 (78.73) | 0.586 (0.245 -1.401) | 0.229 |
| Current regimen of ART |  |  |  |  |  |
| TDF + 3TC + EFV/NVP | 226 (55.53) | 83 (59.71) | 143 (53.36) | 1.000 (Reference) |  |
| AZT + 3TC + EFV/NVP | 85 (20.88) | 33 (23.74) | 52 (19.40) | 1.093 (0.654 -1.827) | 0.733 |
| LPV/r+ 3TC + AZT/TDF | 93 (22.85) | 22 (15.83) | 71 (26.49) | 0.534 (0.308 -0.925) | **0.025*** |
| Other | 3 (0.74) | 1 (0.72) | 2 (0.75) | 0.861 (0.077 -9.646) | 0.904 |
| DR to NRTIs |  |  |  |  |  |
| No | 272 (66.83) | 94 (67.63) | 178(66.42) | 1.000 (Reference) |  |
| Yes | 135 (33.17) | 45 (32.37) | 90 (33.58) | 0.947(0.612 -1.465) | 0.806 |
| DR to NNRTIs |  |  |  |  |  |
| No | 61 (14.99) | 25 (17.99) | 36 (13.43) | 1.000 (Reference) |  |
| Yes | 346 (85.01) | 114 (82.01) | 232 (86.57) | 0.708(0.405-1.235) | 0.224 |
| DR to PIs |  |  |  |  |  |
| No | 357 (87.71) | 123 (88.49) | 234 (87.31) | 1.000 (Reference) |  |
| Yes | 50 (12.29) | 16 (11.51) | 34 (12.69) | 0.895 (0.475 -1.686) | 0.732 |
| Subtype |  |  |  |  |  |
| CRF07_BC | 396 (97.30) | 133 (95.68) | 263 (98.13) | 1.000 (Reference) |  |
| CRF01_AE | 4 (0.98) | 3 (2.16) | 1 (0.37) | 5.932 (0.611 -57.580) | 0.125 |
| B | 2 (0.49) | 0 (0.00) | 2 (0.75) | 0.000 (0.000 -Inf) | 0.982 |
| Other | 5 (1.23) | 3 (2.16) | 2 (0.75) | 2.966 (0.490 -17.968) | 0.237 |

**P* value< 0.1
